# Hemodynamic Support Using Percutaneous Transfemoral Impella 5.0 and Impella RP for Refractory Cardiogenic Shock

**DOI:** 10.1155/2019/4591250

**Published:** 2019-01-23

**Authors:** Pratik K. Dalal, Amy Mertens, Dinesh Shah, Ivan Hanson

**Affiliations:** ^1^Department of Cardiovascular Medicine, Beaumont Health System, Royal Oak, MI, USA; ^2^Oakland University William Beaumont School of Medicine, Royal Oak, MI, USA

## Abstract

Acute myocardial infarction (AMI) resulting in cardiogenic shock continues to be a substantial source of morbidity and mortality despite advances in recognition and treatment. Prior to the advent of percutaneous and more durable left ventricular support devices, prompt revascularization with the addition of vasopressors and inotropes were the standard of care in the management of this critical population. Recent published studies have shown that in addition to prompt revascularization, unloading of the left ventricle with the placement of the Impella percutaneous axillary flow pump can lead to improvement in mortality. Parameters such as the cardiac power output (CPO) and pulmonary artery pulsatility index (PAPi), obtained through pulmonary artery catheterization, can help ascertain the productivity of right and left ventricular function. Utilization of these parameters can provide the information necessary to escalate support to the right ventricle with the insertion of an Impella RP or the left ventricle with the insertion of larger devices, which provide more forward flow. Herein, we present a case of AMI complicated by cardiogenic shock resulting in biventricular failure treated with the percutaneous insertion of an Impella RP and Impella 5.0 utilizing invasive markers of left and right ventricular function to guide the management and escalation of care.

## 1. Introduction

Acute myocardial infarction (AMI) complicated by cardiogenic shock (AMI-CS) is associated with in-hospital mortality between 33% and 55% [1]. Rapid hemodynamic evaluation and left ventricular hemodynamic support using the Impella (Abiomed, Danvers, MA) pumps (2.5, CP, and 5.0) improve outcomes in patients with AMI-CS [2]. However, even with the timely insertion of these devices, mortality in AMI-CS remains unacceptably high [1, 3]. We present a case of AMI-CS in which a patient underwent insertion of Impella CP and revascularization of the infarct-related artery, but remained in refractory shock and required the escalation of hemodynamic support utilizing the percutaneous insertion of Impella RP and Impella 5.0. Herein, clinical decision-making and technical challenges of this approach are outlined.

## 2. Case

A 61-year-old man presented with 2 days of progressively worsening chest pain. Blood pressure was 90/60 mmHg. The 12-lead ECG revealed sinus tachycardia with a rate of 110 bpm and new left bundle branch block. An echocardiogram revealed a left ventricular ejection fraction of 10%, without evidence of mechanical complications. Troponin was 11 ng/mL. In the emergency department, he developed worsening shock and pulmonary edema necessitating mechanical ventilation. He was urgently triaged to the catheterization laboratory.

**Figure 1:**
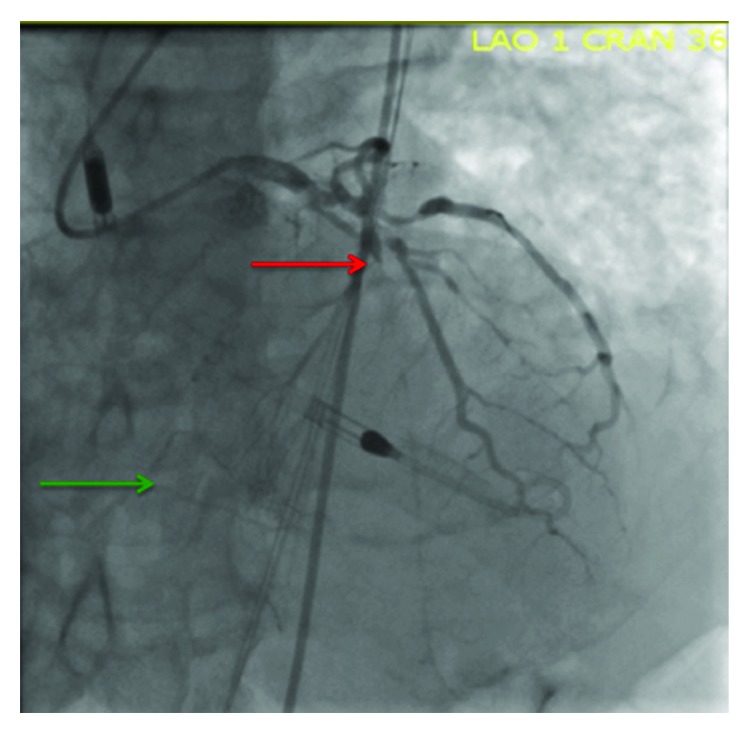
Coronary angiography demonstrating an occluded LAD (red arrow) with collaterals to the RCA (green arrow).

Femoral angiography revealed no evidence of atherosclerosis and femoral artery diameters of 9 mm. An Impella CP was inserted via the left femoral artery, and coronary angiography/intervention was performed via the right femoral artery. Coronary angiography revealed 70% stenosis of the distal left main coronary artery, chronic total occlusion of the left anterior descending artery, 80% calcific stenosis of the left circumflex, and chronic total occlusion of the right coronary artery (Figure 1). Invasive hemodynamics revealed refractory cardiogenic shock and biventricular failure (Table 1). Right ventricular failure was presumed to be due to collateral insufficiency to the chronically occluded right coronary artery.

Given marginal hemodynamics and the presence of right ventricular failure, an RP Impella was inserted via the right femoral vein. Despite adequate flow from the RP (4.7 L/m) and CP (3.5 L/m), hemodynamics only modestly improved (Table 2). Percutaneous revascularization of the culprit severe stenosis in the distal left main and proximal circumflex arteries was challenging but ultimately successful using rotational atherectomy and implantation of a 4.0 × 38 Promus Premiere (Boston Scientific, Marlborough, MA) drug-eluting stent, guided by intravascular ultrasound.

Despite biventricular Impella support using CP and RP catheters and successful revascularization, the patient had persistent cardiogenic shock. This manifested as a markedly reduced cardiac power output (CPO) (Table 2). It was elected to escalate left ventricular support using Impella 5.0. Given the large caliber of the femoral arteries and lack of calcification, percutaneous femoral insertion was performed.

**Figure 2:**
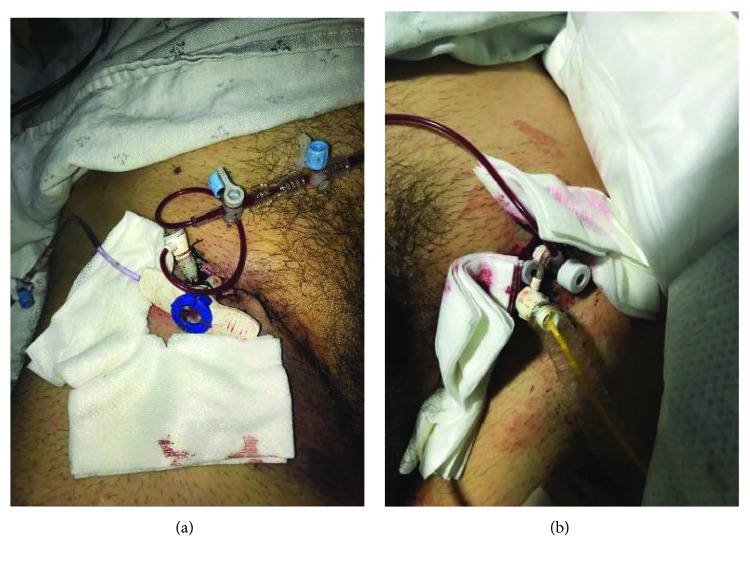
(a) Receiving limb of the external left femoral artery to right superficial femoral artery bypass. (b) Donor limb of the external left femoral artery to right superficial femoral artery bypass.

Anticipating limb ischemia with large bore sheath insertion, an ex vivo bypass circuit was deemed necessary. With ultrasound guidance, an antegrade 5-French sheath was inserted in the right superficial femoral artery. Next, the existing 6-French sheath in the right femoral artery was replaced with progressively larger sheaths to dilate the arteriotomy. Finally, a 23-French sheath (Abiomed, Danvers, MA) was inserted. Via the 23-French sheath, an Impella 5.0 was inserted into the left ventricle and the existing Impella CP was removed through the left femoral artery. Hemodynamics immediately improved (Table 2). Antegrade perfusion of the 14 F left femoral arterial sheath was not undertaken as the caliber of the femoral artery was adequate to accommodate a 4.6 mm sheath. The ex vivo bypass circuit was created by connecting the 14-French left femoral sheath (donor which originally housed the Impella CP) to the 5-French right femoral antegrade sheath (recipient) (Figures 2(a) and 2(b)). Both legs and feet were warm to touch with intact distal pulses.

**Figure 3:**
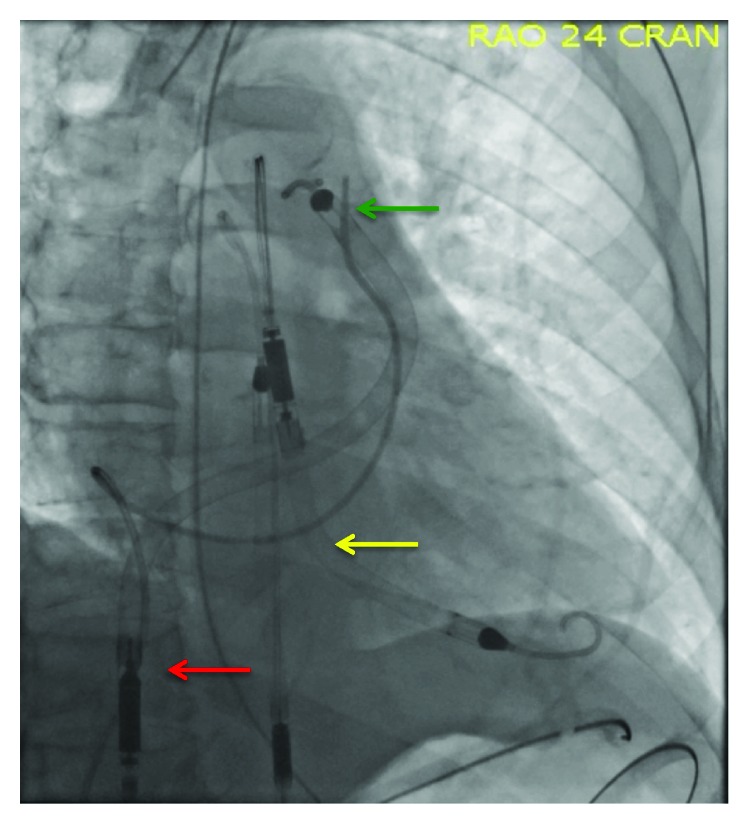
Chest X-ray showing RP Impella with the inlet situated in the inferior vena cava (red arrow) and the outlet in the pulmonary artery (green arrow) and Impella 5.0 with the inlet in the left ventricle and the outlet in the ascending aorta (yellow arrow).

In the cardiac ICU, Impella 5.0 and RP support was maintained (Figure 3). Vasopressors were not required. On hospital day 2, the patient developed profound intravascular hemolysis, transient complete heart block, and pulseless electrical activity requiring cardiopulmonary resuscitation. A repeat echocardiogram revealed small LV and RV cavity sizes. Taken together, the findings suggested that the high flow rate from the Impella 5.0 had caused suction of endocardial tissue, leading to hemolysis and increased vagotonia. This improved with volume resuscitation and blood transfusions with reduction in Impella flow rate. On hospital day 3, the Impella RP was explanted uneventfully. The Impella 5.0 was explanted in the OR. Surgical repair of the right common femoral artery was uneventful. Despite confirmation of antegrade flow beyond the femoral artery, fasciotomy of the anterior compartment of the right leg was performed because of elevated compartment pressure and clinical evidence of limb ischemia. There was evidence of hematoma and myonecrosis in the right thigh, which was treated with resection of the affected muscle. The patient slowly recovered and was discharged to inpatient rehabilitation on hospital day 30. Left ventricular ejection fraction improved to 40%. He was discharged home from rehabilitation on hospital day 60.

## 3. Discussion

Early identification of cardiogenic shock and rapid institution of left ventricular mechanical circulatory support prior to primary PCI result in favorable outcomes. The Detroit Cardiogenic Shock Initiative, in which patients with AMI and cardiogenic shock were supported with Impella prior to PCI so as to minimize the “door to unload time,” resulted in survival to device explant of 89% and survival to discharge of 84% [2]. This represents substantial improvement compared to the historic survival rate of 50% prior to the institution of such an algorithm. Unfortunately, some patients still have refractory shock despite early mechanical support and successful revascularization. The present case illustrates such a patient, who had refractory shock due to both profound LV failure and concomitant RV failure.

### 3.1. Refractory Shock in AMI due to LV Failure despite LV Mechanical Circulatory Support

Left ventricular dysfunction is the cause of shock in ~75% of patients with AMI-CS, with the LAD being the most common infarct-related artery in these cases [4, 5]. A retrospective analysis of the SHOCK Trial (Should We Emergently Revascularize Occluded Coronaries for Cardiogenic Shock) demonstrated that cardiac power output (CPO, calculated as the product of mean arterial blood pressure and cardiac output, divided by 451) is the most potent predictor of survival in AMI-CS [6]. Furthermore, Impella 2.5 (providing up to 2.5 liters per minute of forward flow) increases CPO to a greater degree than IABP, a device that has not been shown to improve survival in AMI-CS [7]. Therefore, we believe that CPO is a reasonable therapeutic target in AMI-CS, and measures to increase CPO to greater than 0.6 should be undertaken.

Impella CP was designed to provide up to 4.0 liters per minute of forward flow, and is now the standard mechanical circulatory device at our institution for the treatment of AMI-CS. However, some patients have refractory shock despite early use of Impella CP and coronary revascularization. If the cause of refractory shock is LV failure, escalation of LV support should be considered. Options include (1) implantation of a durable left ventricular assist device (LVAD), (2) venoarterial extracorporeal membrane oxygenation (VA-ECMO) with a left ventricular vent, or (3) use of a larger axial flow pump, such as Impella 5.0 [8–10]. Of course, escalation of care assumes that multiorgan failure and anoxic brain injury are not present (in which case, a frank discussion with family members about treatment goals and expectations and terminal weaning of support may be most appropriate).

Impella 5.0 is designed to provide up to 5 liters per minute of forward flow. Full LV support provides complete unloading of the left ventricle, which improves coronary blood flow to the culprit and nonculprit territories [11, 12]. Additional forward flow improves blood pressure and CPO and lessens the requirement of vasopressor agents, which have been shown to worsen survival in AMI-CS [13]. Although the size of the catheter is 9 French, the inflow cage is 21 French. Accordingly, device insertion may not be straightforward, and it usually requires surgical cutdown with anastamosis of a graft conduit to the access artery. Alternatively, a sheathless percutaneous insertion can be performed in the femoral or axillary artery [14]. Transcaval insertion allows for the insertion of a large-bore introducer into the femoral vein, then passage through an iatrogenic fistula from the inferior vena cava into the aorta. When Impella support is no longer needed, the introducer sheath can be removed and the fistula occluded by the insertion of a nitinol occluder device [15]. In our case, the femoral arteries were felt to be large enough to accommodate a 23-French Abiomed sheath. This sheath was selected because it features a softer hemostatic valve to allow the passage of the bulky inflow cage of the Impella 5.0 without kinking the catheter shaft or damaging the internal catheter electronics. It is a peel-away sheath, but it was left in place since removal of the sheath would undoubtedly cause profuse bleeding given the mismatch between the sheath size (23 French) and the catheter size (9 French). Certainly, removal of the peel-away sheath could lead to hematoma formation and compartment syndrome. Ex vivo arterial bypass was needed since the large-bore sheath was flow occlusive to the right lower extremity. Techniques for creating the bypass circuit are described elsewhere [16, 17].

### 3.2. Refractory Shock in Anterior AMI with Concomitant RV Failure

Pulmonary artery pulsatility index (PAPi, calculated as pulmonary artery systolic pressure − pulmonary artery diastolic pressure/RA pressure; values < 0.9 suggest RVD) was shown to be a potent predictor of outcome in AMI-CS due to RV failure [18]. In a retrospective analysis of the SHOCK registry, 37% of the patients had hemodynamic evidence of RV dysfunction when presenting with cardiogenic shock. Additionally, in 33% of the patients with RV dysfunction, the infarct-related artery was the LAD [19]. Right ventricular failure in the setting of AWMI could be provoked by collateral insufficiency to a previously occluded right coronary artery, preexisting RV dysfunction from a noncardiac cause, or multivessel coronary occlusion. However, animal models also suggest abnormalities in signaling pathways, such as cell chemotaxis, regulation of endothelial cell proliferation, regulation of apoptosis, and regulation of cytoskeleton organization and cell adhesion in the right ventricle when there is ligation of the left anterior descending artery [20, 21]. Therefore, infarction in one myocardial territory may have additional deleterious effects remotely in distant cardiac tissue. Although the unloading of the left ventricle should reduce pulmonary artery pressure and decrease the work of the RV, there are several mechanisms which lead to a perturbation of RV function. Increased cardiac output from the LVAD increases venous return to the RV, potentially worsening the preexisting RV failure [22]. Excessive leftward shift of the intraventricular septum may also decrease septal contribution to RV contraction, leading to RVF [23, 24]. LV unloading from an LVAD typically reduces tricuspid regurgitation (TR) through decreased RV afterload [25]. In the setting of an incompetent valve, increased RV volume and tethering of valve leaflets to a leftward-shifted septum can intensify TR [25, 26].

Impella RP is a 22-French axial flow pump mounted on an 11-French catheter that is inserted peripherally via the femoral vein and provides up to 4.4 L/min of flow from the inferior vena cava into the pulmonary artery (Figure 3). Results from the Recover Right study showed favorable hemodynamic profiles after insertion, manifested by an improvement in the cardiac index, decrease in central venous pressure, and improved survival at 30 days [27]. In patients who are primarily unloaded with a left-sided mechanical heart pump, a decrease in cardiac power output < 0.6 may suggest poor RV function, especially in cases where the pulmonary artery pulsatility index is <0.9. Several other mechanical circulatory support devices are commercially available for the management of biventricular failure. These include VA-ECMO, biventricular TandemHeart pumps, and various combinations of left-sided Impella and TandemHeart or VA-ECMO pumps [28]. TandemHeart requires a transeptal puncture, a 21 F venous cannula, and a 15-19 F arterial cannula, while VA-ECMO requires an LV “vent” in the form of an Impella to mitigate progressive LV failure due to excessive afterload [29–31].

## 4. Conclusion

We present a case of biventricular failure complicating anterior wall AMI successfully treated with full hemodynamic support using Impella 5.0 and Impella RP. Refractory cardiogenic shock despite mechanical circulatory support in AMI is a challenging clinical scenario. Two potential mechanisms for refractory shock in this setting include concomitant RV failure and persistent LV pump failure. An understanding of the pathogenesis, diagnosis, and timely management of these hemodynamic conditions is likely to improve survival.

## Figures and Tables

**Table 1:** 

RA: 18 mmHg	CI: 1.69 L/min/m^2^
PA: 54/34 mmHg, mean: 31 mmHg	PA Sat: 53%
RV: 54/19 mmHg, EDP: 22 mmHg	FA Sat: 95%
PCWP: 31 mmHg	CPO: 0.74
CO: 3.35 L/min	PAPi: 1.1

**Table 2:** 

Pre-RP Impella	Post-RP Impella	Post-Impella 5.0
CPO: 0.52 (normal > 0.6)	CPO: 0.6	CPO: 0.77
PAPi: 0.7 (normal > 0.9)	PAPi: 0.7	PAPi: 1.0
PA saturation: 45%	PA saturation: 43%	PA saturation: 50%

## References

[1] Kolte D., Khera S., Aronow W. S. (2014). Trends in incidence, management, and outcomes of cardiogenic shock complicating ST-elevation myocardial infarction in the United States. *Journal of the American Heart Association*.

[2] O'Neill W., Basir M., Dixon S., Patel K., Schreiber T., Almany S. (2017). Feasibility of early mechanical support during mechanical reperfusion of acute myocardial infarct cardiogenic shock. *JACC: Cardiovascular Interventions*.

[3] Goldberg R. J., Gore J. M., Alpert J. S. (1991). Cardiogenic shock after acute myocardial infarction: incidence and mortality from a community-wide perspective, 1975–1988. *The New England Journal of Medicine*.

[4] Hochman J. S., Boland J., Sleeper L. A. (1995). Current spectrum of cardiogenic shock and effect of early revascularisation on mortality: results of an international registry. *Circulation*.

[5] Hochman J. S., Sleeper L. A., Webb J. G. (1999). Early revascularization in acute myocardial infarction complicated by cardiogenic shock. *The New England Journal of Medicine*.

[6] Fincke R., Hochman J. S., Lowe A. M. (2004). Cardiac power is the strongest hemodynamic correlate of mortality in cardiogenic shock: a report from the SHOCK trial registry. *Journal of the American College of Cardiology*.

[7] O’Neill W. W., Kleiman N. S., Moses J. (2012). A prospective, randomized clinical trial of hemodynamic support with Impella 2.5 versus intra-aortic balloon pump in patients undergoing high-risk percutaneous coronary intervention: the PROTECT II study. *Circulation*.

[8] Leshnower B. G., Gleason T. G., O’Hara M. L. (2006). Safety and efficacy of left ventricular assist device support in postmyocardial infarction cardiogenic shock. *The Annals of Thoracic Surgery*.

[9] Lawler P. R., Silver D. A., Scirica B. M., Couper G. S., Weinhouse G. L., Camp P. C. (2015). Extracorporeal membrane oxygenation in adults with cardiogenic shock. *Circulation*.

[10] Napp L. C., Kühn C., Hoeper M. M. (2016). Cannulation strategies for percutaneous extracorporeal membrane oxygenation in adults. *Clinical Research in Cardiology*.

[11] Aqel R. A., Hage F. G., Iskandrian A. E. (2010). Improvement of myocardial perfusion with a percutaneously inserted left ventricular assist device. *Journal of Nuclear Cardiology*.

[12] Remmelink M., Sjauw K. D., Henriques J. P. S. (2007). Effects of left ventricular unloading by Impella recover LP 2.5 on coronary hemodynamics. *Catheterization and Cardiovascular Interventions*.

[13] Tarvasmaki T., Lassus J., Varpula M. (2016). Current real-life use of vasopressors and inotropes in cardiogenic shock-adrenaline use is associated with excess organ injury and mortality. *Critical Care*.

[14] Nakamura K., Krishnan S. (2017). First-in-man percutaneous transaxillary artery placement and removal of the Impella 5.0 mechanical circulatory support device. *The Journal of Invasive Cardiology*.

[15] Kamioka N., Patel A., Burke M. A., Greenbaum A., Babaliaros V. (2017). Biventricular Impella placement via complete venous access. *Catheterization and Cardiovascular Interventions*.

[16] Merhi W. M., Turi Z. G., Dixon S., Safian R. D. (2006). Percutaneous ex-vivo femoral arterial bypass: a novel approach for treatment of acute limb ischemia as a complication of femoral arterial catheterization. *Catheterization and Cardiovascular Interventions*.

[17] Kizner L., Flottmann C., Horstkotte D., Gummert J. (2016). Bilateral antegrade perfusion of the superficial femoral artery to prevent limb ischaemia during combined use of Impella CP left ventricular assist device and extracorporeal life support. *Interactive Cardiovascular and Thoracic Surgery*.

[18] Korabathina R., Heffernan K. S., Paruchuri V. (2012). The pulmonary artery pulsatility index identifies severe right ventricular dysfunction in acute inferior myocardial infarction. *Catheterization and Cardiovascular Interventions*.

[19] Lala A., Guo Y., Xu J. (2018). Right ventricular dysfunction in acute myocardial infarction complicated by cardiogenic shock: a hemodynamic analysis of the Should We Emergently Revascularize Occluded Coronaries for Cardiogenic Shock (SHOCK) Trial and Registry. *Journal of Cardiac Failure*.

[20] Erdal C., Karakülah G., Fermancı E. (2012). Early biventricular molecular responses to an acute myocardial infarction. *International Journal of Medical Sciences*.

[21] Bussani R., Abbate A., Biondi-Zoccai G. G. L. (2003). Right ventricular dilatation after left ventricular acute myocardial infarction is predictive of extremely high peri-infarctual apoptosis at postmortem examination in humans. *Journal of Clinical Pathology*.

[22] Farrar D. J., Compton P. G., Hershon J. J., Fonger J. D., Hill J. D. (1985). Right heart interaction with the mechanically assisted left heart. *World Journal of Surgery*.

[23] Moon M. R., Bolger A. F., DeAnda A. (1997). Septal function during left ventricular unloading. *Circulation*.

[24] Morgan J. A., Paone G., Nemeh H. W. (2013). Impact of continuous-flow left ventricular assist device support on right ventricular function. *The Journal of Heart and Lung Transplantation*.

[25] Krishan K., Nair A., Pinney S., Adams D. H., Anyanwu A. C. (2012). Liberal use of tricuspid-valve annuloplasty during left-ventricular assist device implantation. *European Journal of Cardio-Thoracic Surgery*.

[26] Lampert B. C., Teuteberg J. J. (2015). Right ventricular failure after left ventricular assist devices. *The Journal of Heart and Lung Transplantation*.

[27] Anderson M. B., Goldstein J., Milano C. (2015). Benefits of a novel percutaneous ventricular assist device for right heart failure: the prospective RECOVER RIGHT study of the Impella RP device. *The Journal of Heart and Lung Transplantation*.

[28] Kuchibotla S., Esposito M. L., Breton C. (2017). Acute biventricular mechanical circulatory support for cardiogenic shock. *Journal of the American Heart Association*.

[29] Koeckert M. S., Jorde U. P., Naka Y., Moses J. W., Takayama H. (2011). Impella LP 2.5 for left ventricular unloading during venoarterial extracorporeal membrane oxygenation support. *Journal of Cardiac Surgery*.

[30] Chaparro S. V., Badheka A., Marzouka G. R. (2012). Combined use of Impella left ventricular assist device and extracorporeal membrane oxygenation as a bridge to recovery in fulminant myocarditis. *ASAIO Journal*.

[31] Jumean M., Pham D. T., Kapur N. K. (2015). Percutaneous bi-atrial extracorporeal membrane oxygenation for acute circulatory support in advanced heart failure. *Catheterization and Cardiovascular Interventions*.

